# Three-dimensional culture of MSCs produces exosomes with improved yield and enhanced therapeutic efficacy for cisplatin-induced acute kidney injury

**DOI:** 10.1186/s13287-020-01719-2

**Published:** 2020-05-27

**Authors:** Jingyuan Cao, Bin Wang, Taotao Tang, Linli Lv, Zhaoying Ding, Zuolin Li, Ruoyu Hu, Qing Wei, Anran Shen, Yuqi Fu, Bicheng Liu

**Affiliations:** 1 Institute of Nephrology, Zhongda Hospital, Southeast University School of Medicine, Nanjing, 210009 Jiangsu Province China; 2 Department of Cardiothoracic Surgery, Zhongda Hospital, Southeast University School of Medicine, Nanjing, 210009 Jiangsu Province China

**Keywords:** Exosomes, Mesenchymal stem cell, Three-dimensional culture, Acute kidney injury

## Abstract

**Background:**

Exosomes derived from mesenchymal stem cells (MSC-exos) have been demonstrated with great potential in the treatment of multiple human diseases including acute kidney injury (AKI) by virtue of their intrinsic cargoes. However, there are major challenges of low yield and the lack of an established biomanufacturing platform to efficiently produce MSC-exos, thereby limiting their therapeutic application. Here, we aimed to establish a novel strategy to produce MSC-exos with a hollow fiber bioreactor-based three-dimensional (3D) culture system and evaluate the therapeutic efficacy of 3D-exosomes (3D-exos) on AKI.

**Methods:**

Mesenchymal stem cells (MSCs) were isolated from fresh human umbilical cord and cultured in two-dimensional (2D) flasks. 2 × 10^8^ MSCs were inoculated into the hollow fiber bioreactor for 3D culture. The culture supernatants were collected every 1 or 2 days for isolating exosomes. Exosomes from 2D (2D-exos) and 3D cultures were characterized by transmission electron microscopy, nanoparticle tracking analysis, and western blotting analysis of exosome markers. The yield of exosomes from 2 × 10^8^ MSCs seeded in 2D and 3D culture system was compared, based on protein quantification. The therapeutic efficacy of 2D-exos and 3D-exos was investigated in a murine model of cisplatin-induced AKI in vivo and in vitro.

**Results:**

3D culture did not significantly change the surface markers of MSCs, as well as the morphology, size, and exosomal markers of 3D-exos when compared to those of 2D-exos. Compared with conventional 2D culture, the 3D culture system increased total exosome production up to 19.4-fold. 3D-exos were more concentrated in the harvested supernatants (15.5-fold) than 2D-exos, which led to a higher exosome collection efficiency of 3D culture system. In vivo*,* both 2D-exos and 3D-exos significantly alleviated cisplatin-induced murine AKI evidenced by improved renal function, attenuated pathological changes of renal tubules, reduced inflammatory factors, and repressed T cell and macrophage infiltration. Impressively, 3D-exos were more effective than 2D-exos. Moreover, 3D-exos were taken up by tubular epithelial cells (TECs) with improved efficiency, thereby exhibiting superior anti-inflammatory effect and improved viability of TECs in vitro.

**Conclusions:**

In summary, our findings demonstrate that the hollow fiber 3D culture system provides an efficient strategy for the continuous production of MSC-exos which has enhanced therapeutic potential for cisplatin-induced AKI.

## Introduction

Mesenchymal stem cells (MSCs), also known as mesenchymal stromal cells, are characterized by their abilities of self-renewal, differentiation, immunomodulatory and trophic support [1–3], which endows them with enormous potential to treat multiple human diseases, including graft-versus-host disease, systemic lupus erythematosus, Crohn’s disease, cardiovascular and kidney diseases [3–5]. However, safety issues regarding MSC-based therapy, such as embolism, immunogenicity, and potential risk of proliferation, are main concerns for their usage [6–9].

Exosomes, a major class of extracellular vesicles (EVs) that are 30 to 150 nm in diameter, are released by almost all kinds of cells and play important roles in both physiological and pathological conditions [10]. They provide a short- to long-distance form of intercellular communication by shuttling bioactive molecules, including DNA fragments, mRNAs, non-coding RNAs, proteins, and lipids [10, 11]. Very recently, remarkable advances in our understanding of exosomes have spurred a renewed interest in their utility as delivery vehicles for various therapeutic agents ranging from chemotherapeutics to gene therapy [12–14].

An increasing number of studies have demonstrated MSC-derived exosomes (MSC-exos) have innate therapeutic potential by virtue of their intrinsic cargoes. MSC-exos have shown excellent efficacy in tissue repair and regeneration of many organs, including liver, lung, cartilage, myocardium, brain, spinal cord, and kidney [15–22]. Taking acute kidney injury (AKI) as an example, MSC-exos exert a series of renoprotective and regenerative effects through various mechanisms, including anti-inflammatory, immunomodulatory, anti-necroptosis, anti-apoptosis, and promoting cell proliferation [15, 23]. To date, administration of MSC-exos effectively improves clinical outcomes in patients suffering from graft-versus-host disease and chronic kidney diseases [24, 25]. In particular, it is shown that the therapeutic effect of MSC-exos is similar to that of MSCs [1], without the drawbacks of MSC-based therapy, such as unwanted differentiation of engrafted MSCs and embolism. Additionally, MSC-exos possess the characteristic of lower immunogenicity because they lack the antigens on the surface membrane [1, 15]. Therefore, they are likely to represent a novel cell-free therapeutic agent for many human diseases.

However, preclinical and clinical research of exosome-based therapy requires large quantities of MSC-exos. Generally, it requires a dose of 20–200 μg per mouse (10^9^–10^11^ particles) to achieve biological outcomes [26–28]. For clinical testing, one patient needs approximately 100 μg/kg exosomes per treatment [8, 25]. Meanwhile, unlike immortal cells lines, the expansion of MSCs is limited in culture and only cells within passage 6 can be used according to most studies [29, 30]. Therefore, the low yield and the lack of an established biomanufacturing platform to produce sufficient MSC-exos remain major challenges, which currently limit their therapeutic application [8, 15, 31].

In the current study, we reported a novel strategy to produce MSC-exos continuously and efficiently in a hollow fiber bioreactor-based three-dimensional (3D) culture system. Furthermore, we compared the efficacy of the MSC-exos produced from this 3D culture system with those obtained from the conventional 2D flask culture in a murine model of cisplatin-induced AKI.

## Materials and methods

### Culture and identification of human umbilical cord mesenchymal stem cells (hucMSCs)

Primary hucMSCs were donated by Shenzhen Wingor Biotechnology Co, Ltd. In brief, sterile and fresh human umbilical cords were rinsed twice with phosphate-buffered saline (PBS) supplemented with antibiotic-antimycotic (Gibco) to remove cord blood. Then, the umbilical cords were cut into 1-mm^3^-sized pieces and floated in configured media for traditional 2D flask culture. Culture media contained mixed (1:1 ratio) DMEM/F12 (Gibco) and mesenchymal stem cell medium (ScienCell), supplemented with 5% fetal bovine serum (FBS; ScienCell) and 1% antibiotic-antimycotic (Gibco). Half of the medium was replaced every 3 days, and well-developed colonies appeared after 10 days. Early-passage hucMSCs (passages 2–6) were used for downstream experiments.

To detect the typical markers of hucMSCs, the cells of 2D culture (passage 3) and the exfoliated cells from 3D system were incubated with the following monoclonal antibodies: CD29-APC (559883, BD Biosciences), CD34-PE (560941, BD Biosciences), CD44-PE (555479, BD Biosciences), CD45-FITC (555482, BD Biosciences), CD73-PE (561014, BD Biosciences), CD90-PE (561970, BD Biosciences) or with the corresponding isotype. Flow cytometry was performed on BD FACSCalibur (BD Biosciences).

To determine the multidirectional differentiation potential of hucMSCs, the cells of passage 3 were seeded into six-well plates at a density of 1 × 10^5^ cells/well. The culture media were replaced with osteogenic or adipogenic differentiation media (Cyagen Biosciences) in the next day and every 3 days thereafter. Three weeks later, Alizarin Red S and Oil Red O staining were performed to evaluate the extent of osteogenesis and adipogenesis, respectively.

5 × 10^5^ hucMSCs were resuspended in a 15 mL polypropylene tube with 0.5 mL chondrogenic differentiation medium (StemCell technologies). It was capped tightly and centrifuged at 300×*g* for 5 min at room temperature, then the cap was gently loosened, and the tube was incubated at 37 °C with 5% CO_2_. Chondrogenic differentiation medium was replaced every 3 days. Three weeks later, histological sections of the chondrogenic pellet were generated by fixing the pellets in 10% formalin for 30 min, following standard paraffin embedding methods and staining 6 μm sections with Alcian Blue/Nuclear Fast Red.

### 2D culture of hucMSCs

**Fig. 1:**
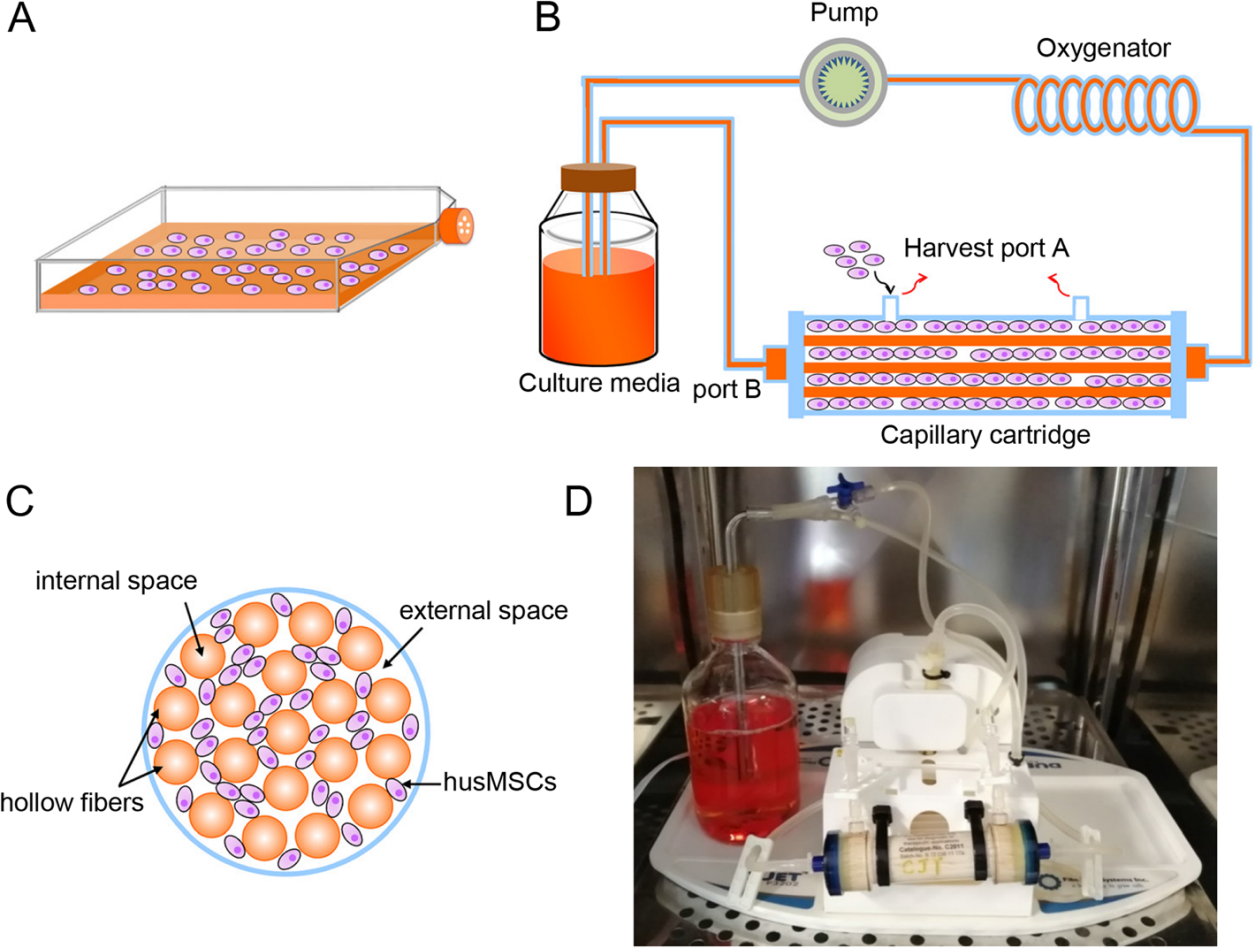
Schematic and photograph of the 2D and 3D culture system. **a** The schematic of conventional 2D flask. **b** The general schematic of the hollow fiber bioreactor-based 3D culture system. The system was composed of a pulsatile perfusion pump, an oxygenator, a cartridge containing thousands of hollow fibers, a bottle of culture medium, and the connecting tube. **c** A general schematic of cross-sectional view through the bioreactor. **d** The photograph of our 3D culture system

HucMSCs were cultured in T225 cm^2^ flasks (Fig. 1a). The glucose concentration of 2D culture media was monitored every 12 h. The culture media were replaced, or cells were passaged when the glucose concentration reduced to 50%. 2 × 10^8^ hucMSCs (passages 4–6) in 2D flasks were washed with PBS and cultured for an additional 48 h in serum-free media. The serum-free culture supernatants were collected for isolating 2D exosomes (2D-exos).

### 3D culture of hucMSCs

The hollow fiber bioreactor FiberCell System (C2011; FiberCell Systems Inc.) was adopted for 3D culture of hucMSCs. This 3D culture system consisted of a pulsatile perfusion pump, an oxygenator, a cartridge containing thousands of hollow fibers, a bottle of culture medium, and the connecting tube. The membrane material of hollow fiber was hydrophilic polysulfone with an internal diameter of approximately 200 μm and a molecular weight cutoff (MWCO) of 20 kDa at 50% retention. The surface area of the hollow fiber bioreactor was 3000 cm^2^ within a volume of 20 mL.

Prior to cell culture, the external and internal spaces of 3D hollow fiber bioreactor were conditioned with PBS or culture media for circulating 24 h, respectively. Then, 2 × 10^8^ hucMSCs within 15 mL suspension were seeded into the external space of 3D culture system. The culture media flowed through the internal space. The flow rate of the pulsatile perfusion pump was 22–28 times per minute.

After 24 h, almost all living cells had attached to the hollow fibers, and the culture media in the external space were replaced with serum-free media. The media in the internal space were still the same with 2D. The glucose concentration in the culture medium bottle was monitored every 12 h and used as a gauge for media replacement. The culture media were replaced immediately when the glucose concentration dropped by 50%. According to the glucose consumption of the 3D culture system, the serum-free supernatants in external space were collected every 24–48 h.

### Trypan blue staining

Cell death in the 2D and 3D systems was determined by trypan blue staining. The cells of 2D system and the exfoliated cells from 3D system were suspended and stained with trypan blue solution (Servicebio). Then, the number of living and dead cells was counted by hemocytometry.

### Isolation and purification of exosomes

The 2D and 3D supernatants were centrifuged at 2000×*g* for 20 min and 13,500×*g* for 30 min at 4 °C to eliminate the cells and debris, followed by filtration with a 0.22-μm filter (Millipore) to remove microvesicles. Then, the supernatants were centrifuged at 200,000×*g* for 120 min (Type 70 Ti rotor, Beckman Coulter Optima L-80 XP) at 4 °C to deposit 2D-exos or 3D exosomes (3D-exos). The exosome pellets were resuspended in PBS and filtered using a 0.22-μm filter once again. The resuspended exosomes were concentrated using 100 KDa MWCO (Millipore) at 4000×*g* for 30 min. Finally, purified 2D-exos and 3D-exos were harvested in 200–400 μL of PBS and stored at − 80 °C.

### Transmission electron microscopy (TEM)

The exosome pellets were fixed with 2.5% glutaraldehyde and post-fixed with 1% osmium for 1 h at room temperature. Samples were then dehydrated with a graded series of ethanol solutions (30%, 50%, 70%, 90%, and 100%) for 15 min each. All samples were infiltrated with and embedded in Epos 812 resin. After polymerization, the coverslips were removed from the bottom of the sample blockers then processed the samples by 70-nm-thick ultrathin sectioning. Every section was double-stained with uranyl acetate and lead citrate [32]. After air drying, specimens were viewed and photographed with TEM (FEI Tecnai G2 Spirit).

### Nanoparticle tracking analysis (NTA)

The particle size of exosomes was measured by NTA with ZetaView PMX 110 (Particle Metrix). Samples were appropriately diluted using PBS, and NTA measurements were recorded and analyzed at 11 positions. The ZetaView system was calibrated using 110 nm polystyrene particles.

### Comparisons of 2D and 3D exosomal production

The exosomes were quantified by protein quantification using the BCA protein assay kit, according to the manufacturer’s instructions (ThermoFisher). To compare the yield of exosomes derived from 2 × 10^8^ hucMSCs seeded in 2D and 3D culture systems, both the total output and the concentration of exosomes per mL of supernatant were assessed.

### Animal models and therapeutic experiments

Adult male C57BL/6 N mice (21–24 g body weight) were purchased from Beijing Vital River Laboratory Animal Technology Co., Ltd., China. Experiments described in this manuscript used protocol approved by the Animal Experimentation Ethics Committees of Southeast University (No. 20190410016). Mice were fed in a specific pathogen-free animal facility with a 12-/12-h light/dark cycle and free access to food and water. For the cisplatin (Cis)-induced injury, a single dose of 18 mg/kg cisplatin was injected intraperitoneally. At 24 h and 48 h after cisplatin injection, mice were injected intravenously with PBS, 2D-exos (100 μg), or 3D-exos (100 μg) (*n* = 6–7, respectively) (Fig. 5a). Mice were euthanized at 96 h post cisplatin injection.

### Renal function and histology

The renal function of mice was monitored by serum creatinine (sCr), which was measured with commercial kits (Jiancheng) according to the manufacturer’s instruction. The kidney tissues collected for histology analysis were fixed with 4% formaldehyde and embedded in paraffin for periodic acid-Schiff (PAS) staining. Tissue damage was scored, based on the percentage of damaged tubules: 0, no damage; 1, < 25%; 2, 25~50%; 3, 50~75%; 4, > 75% [33].

### Immunohistochemistry and immunofluorescence staining

For immunohistochemistry analysis, the paraformaldehyde-fixed and paraffin-embedded kidney sections were incubated with primary antibodies to CD3 (ab16669, Abcam) or CD68 (ab955, Abcam), overnight at 4 °C. Then, the reaction was monitored with an ultrasensitive streptavidin peroxidase detection system (Maixin), which contained secondary goat anti-mouse or anti-rabbit antibody. The sections were then counterstained with hematoxylin. Immunofluorescence staining of paraformaldehyde-fixed kidney sections were performed with primary antibody against KIM-1 (MA5-28211, Invitrogen), followed by incubation with a secondary antibody. Cell nuclei were stained with DAPI. Immunostained sections were visualized under a confocal microscope (FV1000, Olympus).

### Quantitative real-time polymerase chain reaction (PCR) assay

The total RNA from cells or kidney cortex was extracted using the RNAiso plus reagent (Takara), and cDNA was then synthesized using PrimeScript RT reagent kit (Takara) according to the instructions. Quantitative real-time PCR was performed using a 7300 real-time PCR System (Applied Biosystems) to determine the levels of TNF-α, MCP-1, IL-6, and IL-1β. The relative expression levels of mRNA were normalized by β-actin. Primers were synthesized by Generay (Generay Biotech Co., Ltd.). All the primer sequences were listed in Supplementary Table 1.

### Labeling of MSC-exos

To obtain 1,1′-dioctadecyl-3,3,3′,3′-tetramethylindocarbocyanine perchlorate (DiI)-labeled exosomes, purified 2D-exos or 3D-exos were incubated in the presence of 5 μL DiI fluorescent dye (V22885, Invitrogen) for 20 min at 37 °C, then resuspended in 20 mL PBS and ultracentrifuged at 200,000×*g* for 2 h to remove free dye. After being washed twice, the labeled exosomes were resuspended in appropriate PBS for subsequent experiments.

### Cell culture and cellular uptake of MSC-exos in vitro

Mouse tubular epithelial cells (TECs) (a gift from Dr. Jeffery B. Kopp, National Institutes of Health) [34] were cultured in DMEM/F12 (Gibco) supplemented with 10% FBS (ScienCell) and 1% penicillin-streptomycin (Gibco). DiI labeled 2D-exos or 3D-exos were incubated with TECs that administered by 2.5 μg/mL cisplatin for 6 h. The cells were fixed in 4% paraformaldehyde. Further, cell nuclei were stained with DAPI nuclear stain. DiI-positive cells were analyzed by flow cytometry (ACEA NovoCyte) or confocal microscopy.

### Cell treatment

TECs were plated in 6-well plates at a density of 2 × 10^5^ cells/well and incubated until they reached approximately 70% confluence for experiment. The cells were cultured in FBS-free medium for 12 h. Further, cells in cisplatin group were treated with 2.5 μg/mL cisplatin for 6 h, and the culture media were replaced with complete culture medium for 24 h. For the 2D-exos or 3D-exos treatment, cells were treated with 2.5 μg/mL cisplatin for 6 h and then incubated with 2D-exos or 3D-exos (15 μg) in complete culture medium for 24 h. The cells in control group were treated with FBS-free medium for 6 h and incubated in complete culture medium for 24 h.

### Cell counting kit-8 assay

Cell viability was determined by the cell counting kit-8 (CCK-8) assay kit (APExBIO Technology LLC). Briefly, TECs were cultured to reach 70–80% confluence in 96-well plates with different interventions. Ten-microliter CCK-8 reagent was added to the medium and incubated for 2 h. The absorbance was detected at 450-nm wavelength.

### Western blotting

The protein lysates from exosomes or kidney tissues were prepared following standard protocols, and the protein content was determined using the BCA protein assay kit. Then, protein samples were separated by Bis-Tris Gel (Invitrogen) and transferred onto PVDF membranes (Millipore). Membranes were blocked in 5% BSA in TBST for 1 h at room temperature and were incubated with primary antibodies overnight at 4 °C. Then, membranes were washed three times and incubated with secondary antibodies for 2 h at room temperature, and the signals were detected using an ECL advanced system (GE Healthcare). Intensity values expressed as the relative protein expression were normalized to GAPDH (AB2000, Abways). Primary antibodies used were anti-Alix [35] (sc-53540, Santa), anti-CD63 (ab193349, Abcam), anti-CD9 (ab92726, Abcam), and anti-phosphorylated NF-κB p65 (3033, Cell Signaling Technology). Secondary HRP-conjugated antibodies used were anti-mouse IgG and anti-rabbit IgG (Abcam).

### Statistical analysis

Data were expressed as the mean ± SD. Statistical analysis was performed using two-tailed Student’s *t* test or one-way ANOVA. *p* < 0.05 was considered statistically significant.

## Results

### 2D culture and identification of hucMSCs

Optical microscopy showed hucMSCs presented a homogeneous population of spindle fibroblast-like cells (Fig. S1a). When cultured in osteogenic, adipogenic, or chondragenic media, hucMSCs of passage 3 were able to differentiate into osteoblasts, adipocytes or chondroblasts, evidenced by Alizarin Red S staining (Fig. S1b- a), Oil Red O staining (Fig. S1b-b), and Alcian Blue/Nuclear Fast Red staining (Fig. S1b-c), respectively. Flow cytometry analysis revealed that hucMSCs were highly positive (> 97%) for MSC surface markers including CD29, CD44, CD73, and CD90, but negative (< 1%) for hematopoietic stem cell (HSC) surface markers CD34 and CD45 (Fig. S1c). All of these results were consistent with the criteria for defining multipotent MSCs [36].

A schematic diagram of conventional 2D cell culture, for the extraction of 2D-exos, is shown in Fig. 1a. Trypan blue staining showed that the proportion of living hucMSCs (passage 6) was 98%.

### 3D culture and identification of hucMSCs

The general schematic of the hollow fiber bioreactor-based 3D culture system is shown in Fig. 1b. 2 × 10^8^ 2D-cultured hucMSCs were seeded into the external space through port A. The culture medium was continuously perfused into the system through the internal space via the lateral port B. Figure 1c shows a general schematic of cross-sectional view through the bioreactor. Figure 1d shows the photograph of our hollow fiber bioreactor-based 3D culture system.

**Fig. 2:**
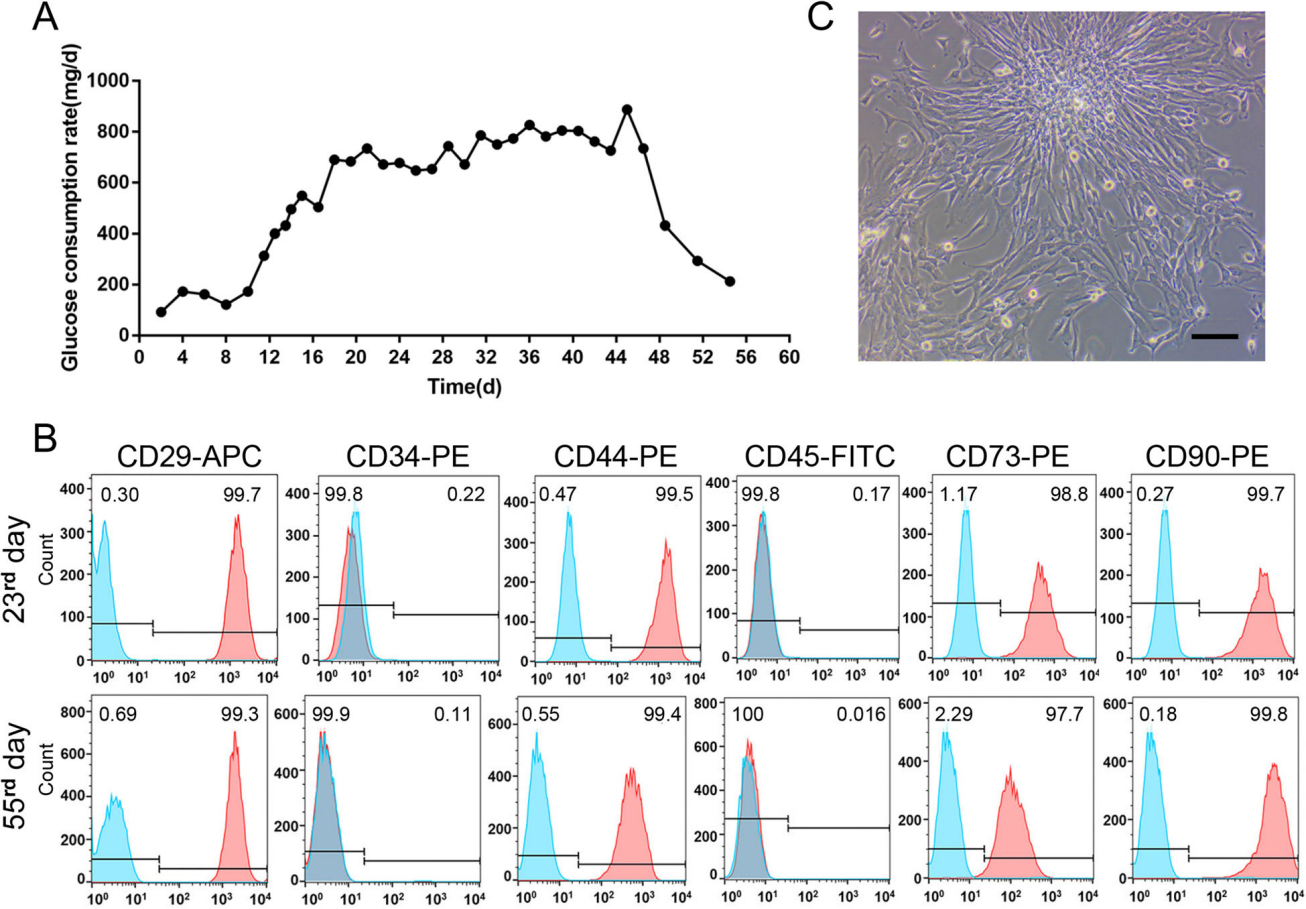
3D Culture of hucMSCs. **a** The glucose consumption rate of 3D culture system. **b** The flow cytometry analysis of MSC (CD29, CD44, CD73, and CD90) and HSC (CD34 and CD45) surface markers of exfoliated cells on the 23rd and 55th days. Blue solid peaks represent the isotype controls, and the red solid peaks represent the marker indicated. **c** The optical micrograph of exfoliated cells in the supernatants from 3D system. Scale bar, 50 μm

This 3D culture system was kept running for a total of 55 days. In the first 10 days, the glucose consumption rate gradually increased, indicating that hucMSCs adapted to the 3D culture system and grew steadily. From the 10th to the 48th day, glucose consumption rate was stable. Thereafter, glucose consumption started to decline (Fig. 2a). Trypan blue staining showed that the living cell ratio of exfoliated hucMSCs from the 3D system was 95% on the 55th day. During the first 10 days, the serum-free supernatants of the external space were collected every 48 h for isolating 3D-exos. After the 10th day, supernatants were collected every 24 h.

To explore whether the phenotype of husMSCs from 3D culture system had changed, on the 10th, 23rd, 34th, and 55th days, the exfoliated cells in the supernatants from the external space were collected and cultured in a 2D culture flask (Fig. 2c). Due to the small number of exfoliated cells, it took 2 weeks of 2D culture to expand optimal cells for MSC and HSC surface markers’ detection. Interestingly, the exfoliated hucMSCs from 3D system were still highly positive (> 97%) for MSC surface markers including CD29, CD44, CD73, and CD90, and negative (< 1%) for CD34 and CD45, suggesting that 3D culture system did not change the phenotype of husMSCs. Figure 2b shows the flow cytometry analysis of surface markers of husMSCs on the 23rd and 55th days.

### Characterization of 2D-exos and 3D-exos

**Fig. 3:**
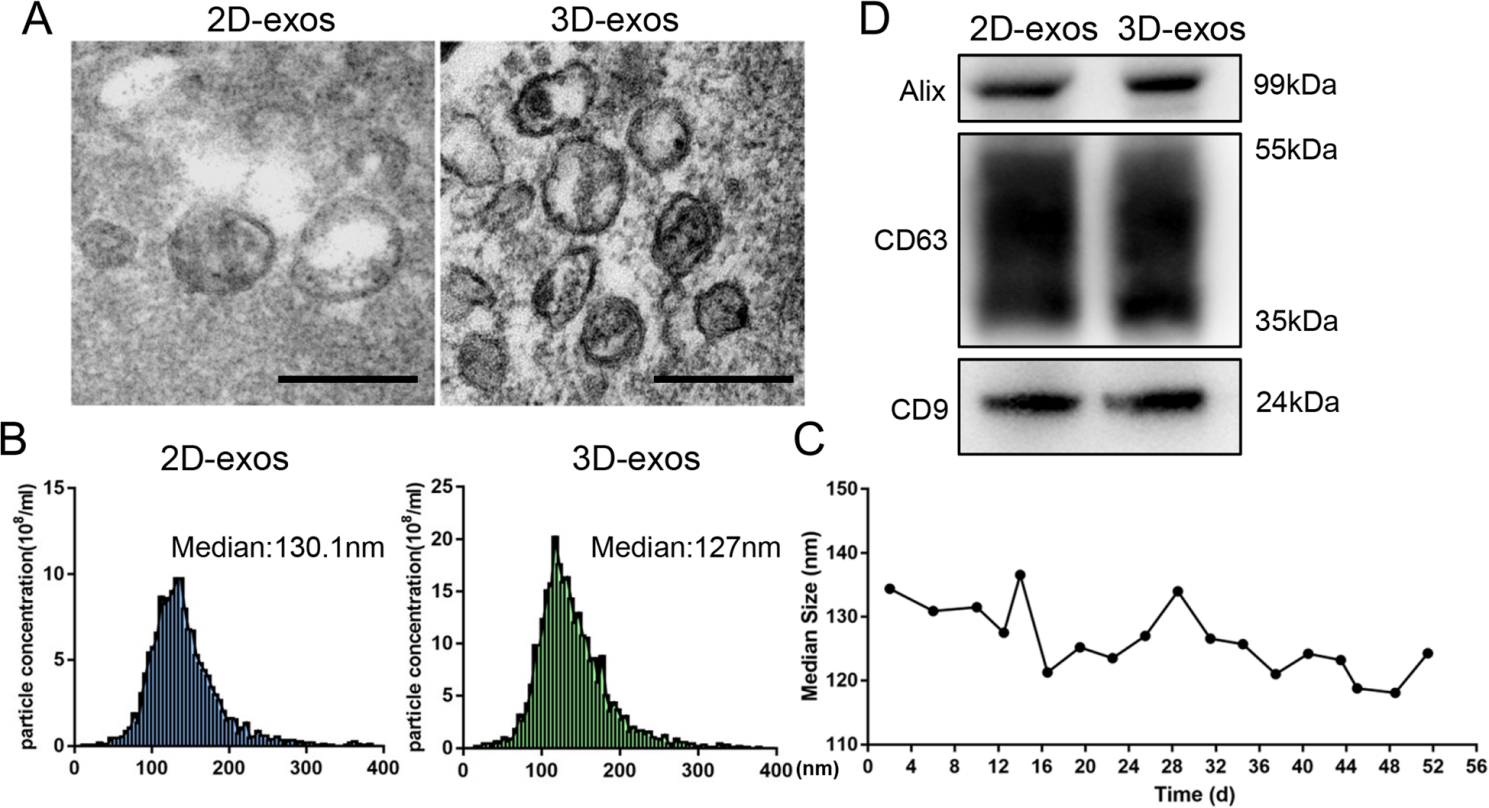
Characterization of 2D-exos and 3D-exos. **a** Morphology of 2D-exos and 3D-exos under TEM. Scale bar, 200 nm. **b** Representative NTA analysis of the particle size distribution of 2D-exos and 3D-exos. The difference between the median diameter of 2D-exos (*n* = 4) and 3D-exos (*n* = 18) was not statistically significant (*p* > 0.05). **c** The particle size distribution of 3D-exos was detected at different time points, and the median diameters of 3D-exos were 118.1–141.3 nm. **d** Western blotting analysis of exosomal surface markers (CD9, CD63, and Alix) of 2D-exos and 3D-exos

TEM showed that 2D-exos and 3D-exos were bilayer membrane vesicles (Fig. 3a). NTA analysis showed that the median diameters of 2D-exos and 3D-exos were 128.4 ± 10.5 nm and 126.3 ± 5.34 nm (Fig. 3b, c), which were consistent with TEM observations. Western blotting showed that exosomal markers, including CD9, CD63, and Alix, were expressed in 2D-exos and 3D-exos (Fig. 3d). These results indicated that there was no significant differences in the morphology, size, or exosomal markers between 2D-exos and 3D-exos.

### High-yield exosome production from 3D culture

2 × 10^8^ hucMSCs were cultured in conventional 2D flasks, and 420 mL supernatants were collected. The same number of hucMSCs was inoculated into 3D culture system, and a total of 525 mL of supernatants were collected within 55 days.

**Fig. 4:**
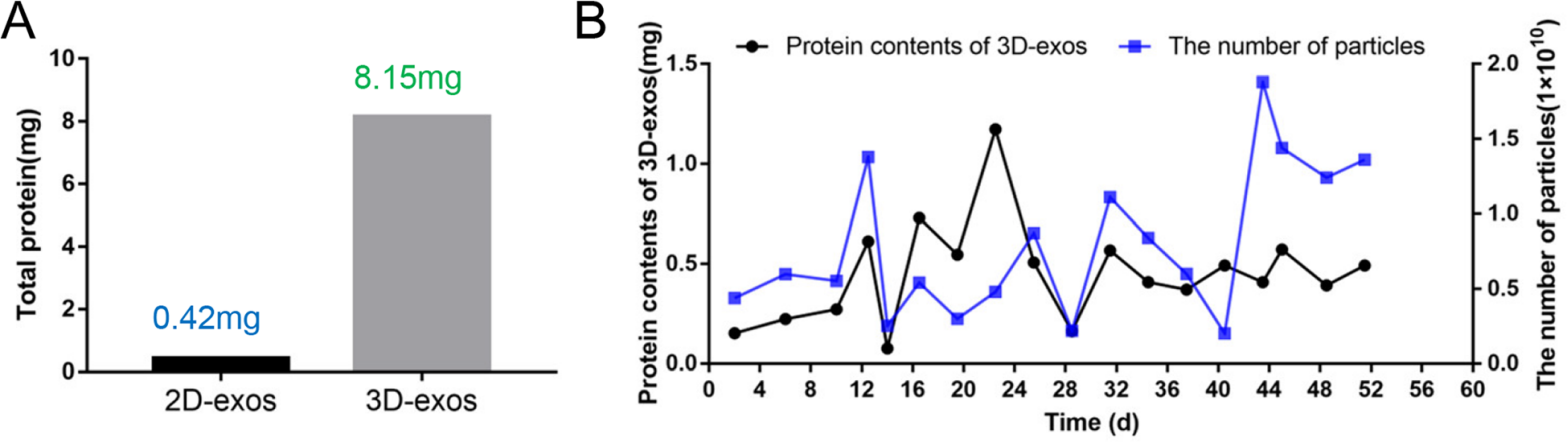
The exosomes yield of 2D-culture and 3D-culture. **a** The total output of exosomes from 2 × 10^8^ MSCs seeded in 2D and 3D culture system. **b** The number of particles and protein contents of 3D-exos harvested from 3D culture system at different time points

Yields of 2D-exos and 3D-exos were detected by protein quantification. The yield of exosomes extracted from 2D-cultured supernatants was 0.42 mg, while that from 3D-cultured supernatants was 19.4-fold higher, with a maximum yield of 8.15 mg (Fig. 4a). 3D-exos were more concentrated in the harvested supernatants (15.5-fold) than 2D-exos, which led to much higher efficiency of isolation through labor-intensive differential ultracentrifugation. Figure 4b shows the number of particles and protein contents of 3D-exos harvested from 3D culture system at different time points.

### Enhanced renoprotective efficacy of 3D-exos in cisplatin-treated mice

**Fig. 5:**
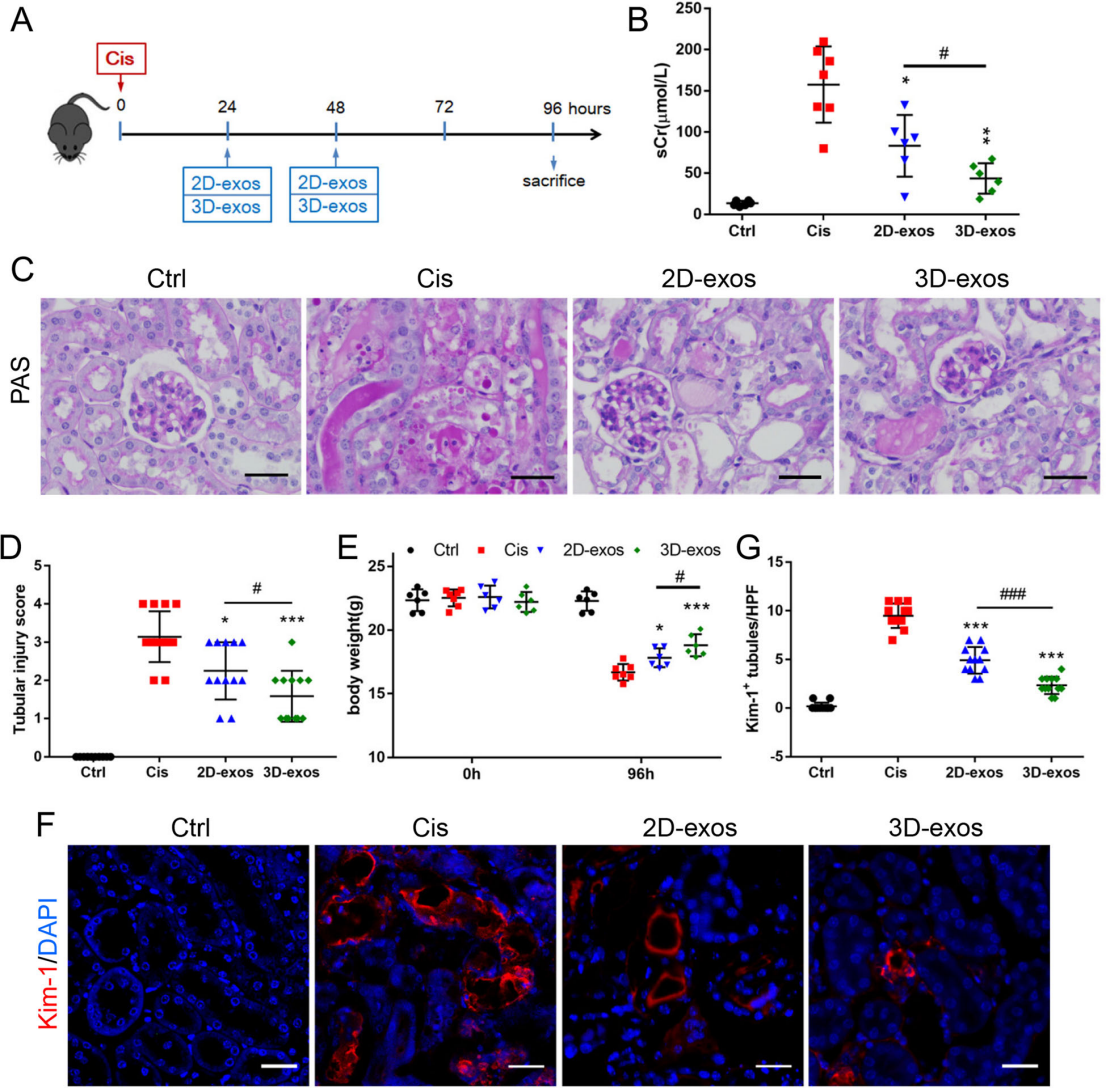
The therapeutic efficacy of 2D-exos and 3D-exos in cisplatin-induced AKI mice model. **a** Schematic diagram of the experimental design. In brief, mice were concurrently treated with PBS, 2D-exos (100 μg) or 3D-exos (100 μg) at 24 h and 48 h after cisplatin injection, and were sacrificed at 96 h after disease induction. **b** Effects of 2D-exos and 3D-exos on serum creatinine (*n* = 6–7). **c** Representative images of PAS staining of renal cortex. Scale bar, 50 μm. **d** The quantification of tubular injury based on PAS staining (*n* = 6–7). **e** Effects of 2D-exos and 3D-exos on the body weight (n = 6–7). **f** Representative confocal images of kidney injury molecular-1(Kim-1) in tubules. Scale bar, 25 μm. **g** The quantification of Kim-1^+^ tubules per HPF (*n* = 6). Data are presented as mean ± SD, *p < 0.05, **p < 0.01, ***p < 0.001 vs. Cis group, ^#^*p* < 0.05, ^###^*p* < 0.001

To compare the therapeutic efficacy of 2D-exos and 3D-exos, a murine model of cisplatin-induced AKI was established, and the treatment protocol was described in detail in the “Materials and methods” section (Fig. 5a). Treatment with 3D-exos caused a more obvious reduction in the level of sCr compared with 2D-exos (Fig. 5b). Mice treated with cisplatin showed extensive epithelial cell swelling, vacuolar degeneration, necrosis, detachment, and casts’ formation occurring predominantly in the proximal tubules, all of which were markedly attenuated by treatment with either 2D-exos or 3D-exos. However, 3D-exos-treated group improved more significantly (Fig. 5c, d). The mouse body weight of 3D-exos-treated group was significantly higher than that of 2D-exos-treated group (Fig. 5e). Besides, kidney sections were stained with anti-KIM-1 antibody for detecting injured tubules. The results demonstrated that KIM-1 was expressed lower in 3D-exos-treated group (Fig. 5f, g). Collectively, these data clarified that both 2D-exos and 3D-exos can alleviate cisplatin-induced murine AKI, but 3D-exos were more effective.

### Enhanced anti-inflammatory efficacy of 3D-exos in cisplatin-treated mice

**Fig. 6:**
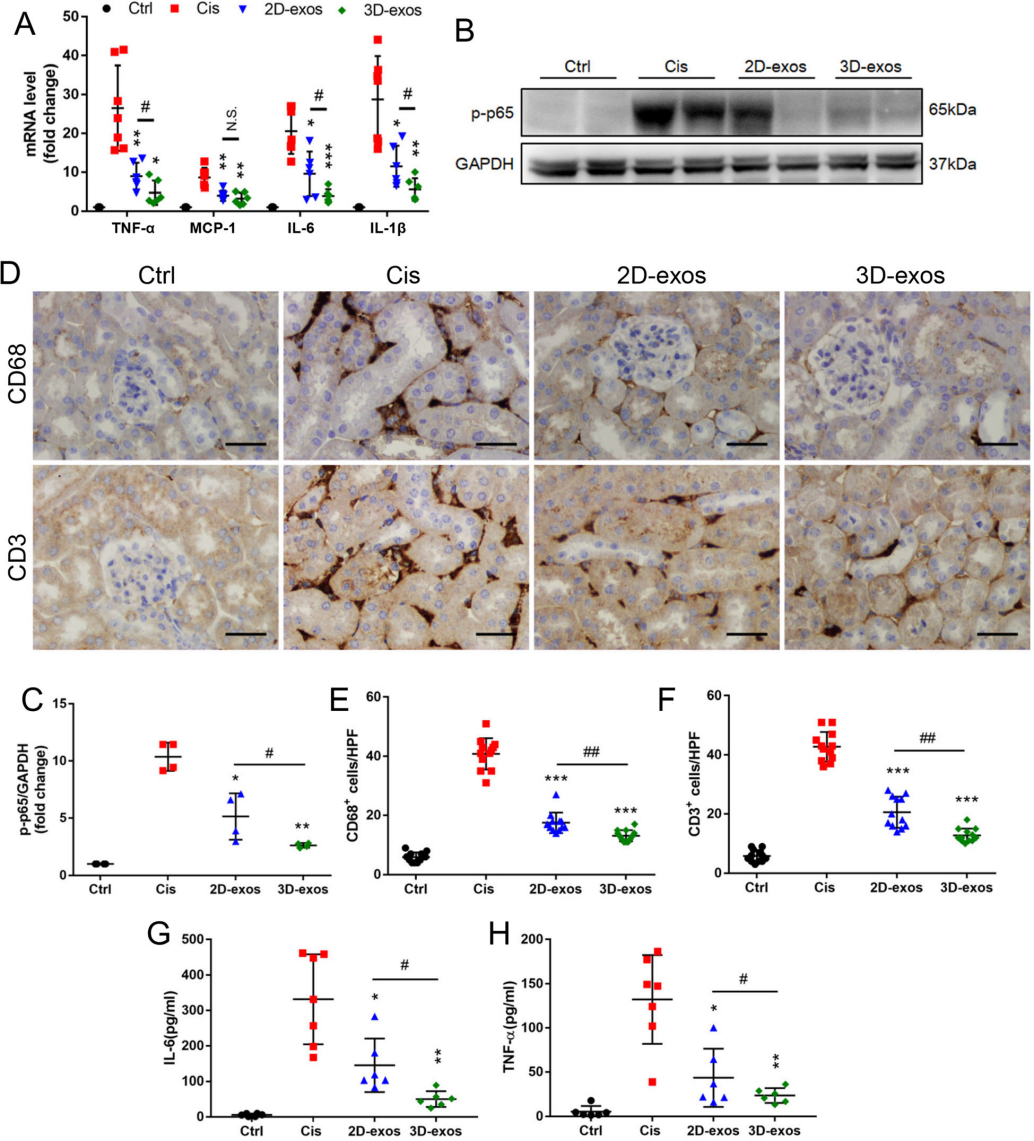
Enhanced anti-inflammatory efficacy of 3D-exos in cisplatin-treated mice. **a** Real-time PCR analysis of inflammatory cytokine mRNA levels in kidney tissues (*n* = 6–7). **b** Western blotting analysis of p-p65 in kidney tissues. **c** Quantification of p-p65 in kidney tissues (*n* = 4). **d** Representative immunostaining images of CD68^+^ macrophages or CD3^+^ T cells in the tubulointerstitium. Scale bar, 50 μm. **e** Quantification of CD68^+^ macrophages in the tubulointerstitium (*n* = 6). **f** Quantification of CD3^+^ T cells in the tubulointerstitium (*n* = 6). **g** Levels of IL-6 in the mouse serum was detected by ELISA (*n* = 6–7). **h** Levels of TNF-α in the mouse serum was detected by ELISA (*n* = 6–7). Data are presented as mean ± SD, *p < 0.05, **p < 0.01, ***p < 0.001 vs. Cis group, ^#^*p* < 0.05, ^##^*p* < 0.01

To fully assess the treatment efficacy of 2D-exos and 3D-exos, we also measured the mRNA expressions for TNF-α, MCP-1, IL-6, and IL-1β, which showed decreased expression after 3D-exos treatment (Fig. 6a), accompanied by decreased expression of NF-κB p-p65 (Fig. 6b, c). In addition, the kidney interstitial infiltration of inflammatory cells, such as macrophages (CD68 positive) and T cells (CD3 positive), was significantly diminished by 3D-exos treatment (Fig. 6d–f). These inflammatory mediators were also blunted with 2D-exos, however, to a lesser extent than that with 3D-exos. Meanwhile, levels of IL-6 and TNF-α in mouse serum of 3D-exos-treated group were lower than 2D-exos-treated group (Fig. 6g, h). These data suggested the anti-inflammatory efficacy of 3D-exos was significantly higher than that of 2D-exos.

### Enhanced therapeutic efficacy of 3D-exos in cisplatin-treated TECs

**Fig. 7:**
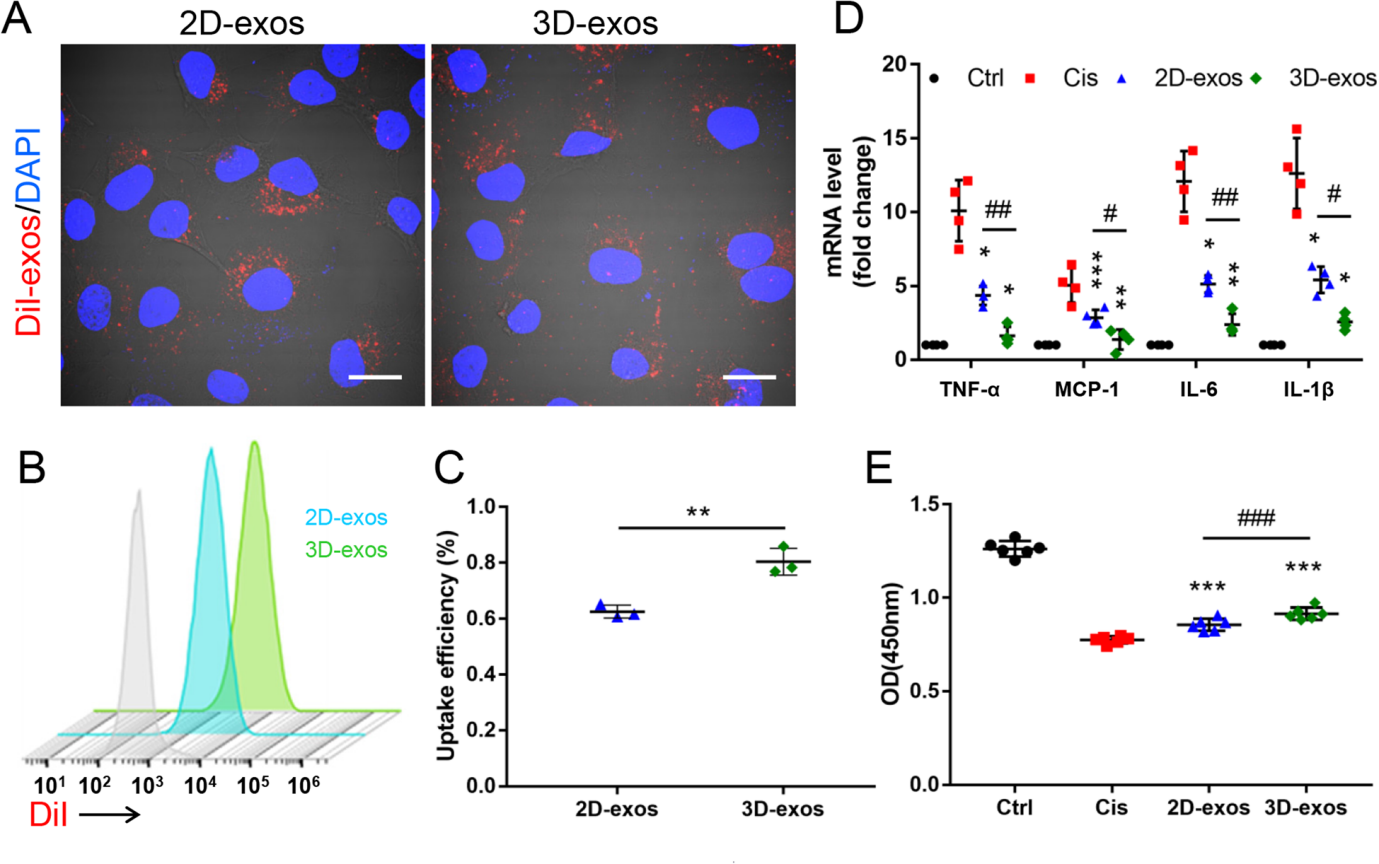
Enhanced therapeutic efficacy of 3D-exos in cisplatin-treated TECs. **a** TECs took up 2D-exos and 3D-exos. DiI-positive (red) cells were observed under confocal microscope. Scale bar, 10 μm. **b** DiI-positive cells detecting by flow cytometry. Blue solid peak represents cisplatin-treated TECs internalizing DiI-labeled 2D-exos, and the green solid peak represents cisplatin-treated TECs internalizing DiI-labeled 3D-exos. **c** The uptake efficiency of 2D-exos and 3D-exos. **d** Real-time PCR analysis of inflammatory cytokine mRNA levels in TECs (*n* = 4). **e** CCK-8 assay in TECs (*n* = 6). Data are presented as mean ± SD, *p < 0.05, **p < 0.01, ***p < 0.001, vs. Cis group, ^#^*p* < 0.05, ^##^*p* < 0.01, ^###^*p* < 0.001

Considering that TECs are extremely sensitive to toxins, protecting TECs from such insults largely determines the prognosis of AKI [37], we speculated that MSC-exos would protect against cisplatin-induced AKI, at least in part, through targeting TECs. Therefore, TECs were used to explore the therapeutic effect of 2D-exos and 3D-exos in vitro. Higher levels of DiI-labeled 3D-exos were found to be internalized by cisplatin-treated TECs (Fig. 7a–c). The uptake efficiency of DiI-labeled 2D-exos and 3D-exos by cisplatin-treated TECs were approximately 60% and 80%, respectively. Furthermore, compared with 2D-exos, 3D-exos markedly inhibited cisplatin-induced mRNA expression of TNF-α, MCP-1, IL-6, and IL-1β (Fig. 7d). Moreover, the cell viability of cisplatin-injured TECs was significantly improved by treatment with 3D-exos (Fig. 7e). Above all, these findings suggested that 3D-exos were more easily taken up by TECs and had a more potent ability to ameliorate cisplatin-induced inflammation and to improve the viability of TECs.

## Discussion

Adult stem cells are extensively used for the treatment of multiple human diseases, with the secretion of soluble factors and EVs that can enhance the repair of damaged tissues through paracrine regulation of local cells [1, 15, 38]. As a major class of EVs, MSC-exos show innate therapeutic potential by virtue of their intrinsic cargoes [15, 19]. MSC-exos represent a sweet spot for cell-based therapies. They can be used as nature’s own delivery tool, act as biotherapeutics, and mimic the action of cellular therapies without the side effects of embolism and proliferation [8]. However, the lack of a platform to obtain sufficient MSC-exos remains a challenge of therapeutic development. In this study, we discovered a novel solution by using a hollow fiber bioreactor-based 3D culture system, which could culture large quantities of husMSCs over a period of 55 days, thereby resulting in a 19.4-fold improvement of MSC-exos yield than that of 2D culture. Furthermore, we explored that 3D-exos showed superior renoprotective efficacy by alleviating inflammation in vivo and in vitro.

3D culture platform presents more advantages over conventional 2D culture. First, this 3D culture system can culture a multitude of cells (estimated to be in the order of 10^9^ cells) within a standard cartridge [39]. Second, high-yield of exosomes can be achieved. Compared with 2D culture, the exosome production of 3D culture is 19.4 times higher. This is mainly due to the fact that the external space of the 3D system fills with serum-free medium, and the supernatants for extracting 3D-exos can be collected continuously. Third, 3D-exos are more concentrated in the harvested supernatants (15.5-fold) than 2D-exos, which leads to a higher exosome collection efficiency. In order to obtain equivalent output from the 3D system, approximately 230 T225 cm^2^ flasks are required to extract 2D-exos. 3D culture reduces the use of plastic consumables such as cell culture flasks and centrifuge tubes. Furthermore, compared with the 2D culture system that requires several operating hours to harvest an equivalent quantity of exosomes, it takes less than 30 min daily in the 3D culture system. In these terms, 3D culture is both human resource-efficient and environmentally friendly.

In recent years, other researchers have used scaffolds, spheroid culture, or microcarrier-based 3D to culture MSCs [40–42]. Previously, Zhang et al. [40] demonstrated that compared with 2D culture, human bone marrow-derived MSCs (hBM-MSCs) seeded in the 3D collagen scaffolds generated more exosomes with improved repair function in rats after traumatic brain injury. They transferred 3 × 10^6^ hBM-MSCs per scaffold into 200 μL of culture medium for exosome isolation, which was harvested only once. Haraszti et al. proved that scalable microcarrier-based 3D cultures could double the density of MSCs and yield more exosomes than 2D cultures [41]. Although these microcarrier-based 3D cultures had a large total surface area (1150 cm^2^) which can culture millions of MSCs, the culture media could only be collected once. In contrast to the other 3D culture systems, hundreds of millions of hucMSCs could be cultured in this hollow fiber bioreactor 3D culture system over a longer time period, which could produce 3D-exos in a continuous and efficient manner.

Despite advances in medical care, AKI remains an independent predictor of in-hospital mortality [43, 44]. Currently, there are no reliable therapies existing to prevent or treat established AKI [44]. MSC-exos may represent a novel cell-free therapeutic agent for AKI. A wealth of researches corroborated that 2D-exos derived from MSCs could ameliorate AKI [45, 46]. Zhou et al. [45] indicated hucMSC-derived exosomes could repair cisplatin-induced AKI through ameliorating oxidative stress and cell apoptosis and promoting cell proliferation in rats. Collino et al. [46] showed EVs derived from bone marrow MSCs protected the host from glycerol-induced AKI by carrying microRNAs. In the present study, we compared the therapeutic efficacy between 2D-exos and 3D-exos in cisplatin-induced AKI and found that 3D-exos exhibited superior renoprotective effects than 2D-exos, probably because 3D-exos were more easily taken up by TECs. Previous studies have shown that EVs could be internalized by clathrin-mediated or clathrin-independent endocytosis, such as macropinocytosis and phagocytosis as well as through endocytosis via caveolae and lipid rafts [47]. The process of endocytosis is complex and depends on the EVs and the recipient cells. Considering that the surface proteins of EVs allow them to be targeted and captured by recipient cells [48], 3D culture may alter exosomal membrane proteins, thus making them more easily be taken up by TECs. Interestingly, one study demonstrated that clathrin could mediate the endocytosis of TECs [49]. Changes in the expression of endocytosis-related proteins in damaged TECs are worthy of attention in the future.

On the other hand, the superior renoprotective ability of 3D-exos was also dependent on the components of their cargos. Yang et al. [50] demonstrated that 195 kinds of miRNAs and proteins, such as neprilysin, insulin-degrading enzyme, and heat shock protein 70, in exosomes derived from hucMSCs cultured in 3D scaffold are dramatically different from those in 2D culture. These 3D-exos exerted enhanced therapeutic effects on ameliorating the memory and cognitive deficits in Alzheimer’s disease mice through their special cargo. The results showed that the content of exosomes was affected by the culture conditions. In future studies, it would be of interest to employ comparative proteomic and RNA-seq to dissect the differences in proteins and nucleic acids between 2D-exos and 3D-exos.

## Conclusions

Taken together, we discovered a novel strategy for producing MSC-exos in a hollow fiber bioreactor-based 3D culture system, which could produce MSC-exos continuously and efficiently. Compared with conventional 2D culture, 3D culture yielded more exosomes and greatly improved the efficiency of exosome collection. Meanwhile, 3D-exos had a stronger renoprotective efficacy in ameliorating cisplatin-induced AKI than 2D-exos. The improved yield and enhanced therapeutic efficacy of 3D-exos make MSC-exos closer to clinical therapy.

## Supplementary information

**Additional file 1: Supplementary Table 1.** Primers used in this study. **Figure S1.** Identification of hucMSCs. **a** HucMSCs exhibited a spindle fibroblast-like morphology. Scale bar: 50 μm. **b** HucMSCs could differentiate into osteoblasts, adipocytes or chondroblasts, evidenced by Alizarin Red S staining (b-a), Oil Red O staining (b-b), and Alcian Blue/Nuclear Fast Red staining (b-c), respectively. Scale bar: 50 μm. **c** Flow cytometry analysis of MSC (CD29, CD44, CD73 and CD90) and HSC (CD34 and CD45) surface markers. Blue solid peaks represent the isotype controls and the red solid peaks represent the marker indicated.

## Data Availability

The datasets supporting the conclusions of this article are included within the article and its additional files.

## Abbreviations

MSC-exos: Exosomes derived from mesenchymal stem cells
AKI: Acute kidney injury
3D: Three-dimensional
3D-exos: 3D-exosomes
2D: Two-dimensional
2D-exos: 2D-exosomes
MSCs: Mesenchymal stem cells
EVs: Extracellular vesicles
hucMSCs: Human umbilical cord mesenchymal stem cells
PBS: Phosphate-buffered saline
MWCO: Molecular weight cut off
NTA: Nanoparticle tracking analysis
Cis: Cisplatin
sCr: Serum creatinine
PAS: Periodic acid-Schiff
PCR: Polymerase chain reaction
DiI: 1,1′-Dioctadecyl-3,3,3′,3′-tetramethylindocarbocyanine perchlorate
TECs: Tubular epithelial cells
CCK-8: Cell counting kit-8
HSC: Hematopoietic stem cell
hBM-MSCs: Human bone marrow-derived MSCs
