# Supplementary material for: Three-dimensional culture of MSCs produces exosomes with improved yield and enhanced therapeutic efficacy for cisplatin-induced acute kidney injury
Source: Stem Cell Res Ther. 2020 May 27;11:206. doi: 10.1186/s13287-020-01719-2 (PMC7251891; doi:10.1186/s13287-020-01719-2)
Supplement: Supplementary file 1 — Additional file 1: Supplementary Table 1. Primers used in this study. Figure S1. Identification of hucMSCs. a HucMSCs exhibited a spindle fibroblast-like morphology. Scale bar: 50 μm. b HucMSCs could differentiate into osteoblasts, adipocytes or chondroblasts, evidenced by Alizarin Red S staining (b-a), Oil Red O staining (b-b), and Alcian Blue/Nuclear Fast Red staining (b-c), respectively. Scale bar: 50 μm. c Flow cytometry analysis of MSC (CD29, CD44, CD73 and CD90) and HSC (CD34 and CD45) surface markers. Blue solid peaks represent the isotype controls and the red solid peaks represent the marker indicated. [file 13287_2020_1719_MOESM1_ESM.zip › Supplementary Table 1 Primers used in this study.docx]

**Supplementary Table 1** Primers used in this study.

| Gene | | Forward | Reverse |
| --- | --- | --- | --- |
| β-actin-MUS | | GGGAAATCGTGCGTGAC | AGGCTGGAAAAGAGCCT |
| MCP-1-MUS | TTGAGGTGGTTGTGGAAAAGG | | GTGCTGACCCCAAGAAGGAAT |
| TNF-α-MUS | AGACAGAGGCAACCTGACCAC | | GCACCACCATCAAGGACTCAA |
| IL-1β-MUS | GGTAAGTGGTTGCCCATCAGA | | GTCGCTCAGGGTCACAAGAAA |
| IL-6-MUS | GTCACCAGCATCAGTCCCAAG | | CCCACCAAGAACGATAGTCAA |
